# Grupos de riesgo por COVID-19 y sus estrategias para enfrentar la sobrecarga informativa en el primer año de la pandemia en Chile

**DOI:** 10.18294/sc.2023.4305

**Published:** 2023-02-04

**Authors:** Verónica Rocamora Villena, Macarena Peña y Lillo, Patricia Junge Cerda, Cecilia Prieto Bravo

**Affiliations:** 1 Doctora en Comunicación Social. Directora, Magíster en Ciencias de la Comunicación, Universidad de Santiago de Chile, Santiago, Chile. veronica.rocamora@usach.cl Universidad de Santiago de Chile Universidad de Santiago de Chile Santiago Chile veronica.rocamora@usach.cl; 2 Doctora en Comunicación. Directora, Magíster en Comunicación, Universidad Diego Portales, Santiago, Chile. macarena.penaylillo@udp.cl Universidad Diego Portales Universidad Diego Portales Santiago Chile macarena.penaylillo@udp.cl; 3 Doctora en Antropología. Profesora adjunta, Departamento de Fonoaudiología, Universidad de Chile, Santiago, Chile. Patricia.junge@uchile.cl Universidad de Chile Departamento de Fonoaudiología Universidad de Chile Santiago Chile Patricia.junge@uchile.cl; 4 Master in Health Inequalities and Public Policy. Candidata a Doctora, University of Edinburgh, Edimburgo, Escocia. clprieto@uc.cl University of Edinburgh University of Edinburgh Edimburgo Escocia clprieto@uc.cl

**Keywords:** Pandemia de COVID-19, Enfermedades Crónicas, Comunicación en Salud, Acceso a la Información, Evitación de Información, Chile, COVID-19 Pandemic, Chronic Disease, Health Communication, Access to Information, Information Avoidance, Chile

## Abstract

Este artículo se enmarca en el proyecto EIS-COVID sobre acceso y uso de información en el contexto de la pandemia de COVID-19 en Chile. Su objetivo fue conocer cómo se constituyó el entorno informativo de las personas en la primera etapa de la pandemia. El artículo muestra los resultados de un estudio cualitativo enfocado en personas pertenecientes a grupos de riesgo por COVID-19: personas mayores de 18 y menores de 65 años con enfermedades crónicas (hipertensión y diabetes) y personas de 65 años y más. Se realizaron 90 entrevistas semiestructuradas en las regiones Metropolitana y de Valparaíso entre septiembre de 2020 y enero de 2021. Se identifica la problemática de la sobrecarga informativa para estos grupos y las estrategias que utilizaron para enfrentarla: a) la evitación de información, b) la corroboración de contenidos y búsqueda activa de fuentes confiables, o c) el uso diferenciado de medios.

## Abstract (en)

As a part of the EIS-COVID project on the access and use of information during the COVID-19 pandemic in Chile, the objective of this paper was to ascertain how people’s informational environment was constructed during the first stage of the pandemic. It discusses the results of a qualitative study of people belonging to risk groups for COVID-19: people over 18 and under 65 with chronic diseases (hypertension and diabetes) and people 65 and over. Ninety semi-structured interviews were conducted in the Metropolitan and Valparaíso regions between September 2020 and January 2021. The results reveal the problematic nature of the information overload encountered by these groups and the strategies they used to navigate it: a) information avoidance; b) content corroboration and active search for reliable sources; and c) differentiated media use.

## INTRODUCCIÓN

“¿*Están ustedes preparados para prevenir o reducir al mínimo la morbilidad y la mortalidad humana, la perturbación social y las consecuencias económicas causadas por una pandemia de influenza*?”^1^

De esta forma nos interpelaba la Organización Mundial de la Salud (OMS), en el año 2005, en un documento que empieza situándonos en un escenario futuro, incierto, en el que una desconocida gripe proveniente de una provincia remota se convierte en una pandemia mundial, generando gran mortandad, crisis económica y el colapso de los sistemas sanitarios. Este material es parte de los numerosos documentos y protocolos que se han elaborado desde el año 2005, en el marco del Reglamento Sanitario Internacional, para la preparación y respuesta ante una posible pandemia de gripe, en la que la comunicación ha tenido un lugar relevante para la gestión del riesgo^2^. Las graves consecuencias sociosanitarias y económicas que ha traído el COVID-19 a nivel mundial dan cuenta de que la mayoría de los países y sus gobiernos no estaban en condiciones de enfrentar una pandemia, mucho menos en un escenario comunicacional que se caracterizó por una circulación de información sin precedentes, al punto de que autoridades sanitarias y expertos sostuvieron que, en conjunto con la pandemia, se desarrolló una infodemia^3^^,^^4^^,^^5^. Dado este contexto comunicacional, cobra especial relevancia comprender la forma en que las personas se han informado durante la pandemia y cómo han lidiado con esta abundancia de información. 

Este artículo se enmarca en el proyecto EIS-COVID (proyecto ANID-COVID0508) sobre acceso y uso de información en el contexto de la pandemia de COVID-19 en Chile, cuyo objetivo general fue conocer cómo se constituyó el entorno informativo de las personas en el contexto de la pandemia, en otras palabras, cuáles eran las múltiples fuentes y mensajes que rodeaban a las personas, estableciendo una relación con la percepción de riesgo y con la adopción de comportamientos preventivos. En el marco de este proyecto, se generaron dos estudios: a) una encuesta a población general mayor de 18 años, y b) un estudio cualitativo enfocado en personas pertenecientes a grupos de riesgo por COVID-19, es decir, personas de 65 años y más o personas mayores de 18 y menores de 65 años, pero que viven con enfermedades crónicas como hipertensión y diabetes. 

Este artículo da cuenta de los resultados del estudio cualitativo que muestra las estrategias que utilizaron las personas pertenecientes a estos grupos de riesgo para sobrellevar la sobrecarga informativa que se experimentó en el primer año de la pandemia por COVID-19. Centrarse en este período adquiere especial relevancia para revisar las lecciones aprendidas y continuar enfrentando esta o futuras crisis sociosanitarias prolongadas y de alta incertidumbre. 

### Breve contexto chileno

En el momento en que la OMS declaró la pandemia por COVID-19, el 11 de marzo de 2020^6^, Chile vivía un particular escenario político debido al estallido social de octubre de 2019, motivado por profundas desigualdades socioeconómicas y las exigencias ciudadanas por realizar transformaciones estructurales urgentes^7^. Las críticas al manejo de esta crisis se vieron reflejadas en una disminución de la aprobación de las autoridades del gobierno que, a esa fecha, alcanzaba solo un 12%^8^, pero la desconfianza de la ciudadanía se extendió a otras instituciones y los medios de comunicación no quedaron exentos.

Si bien la pérdida de credibilidad de los medios de comunicación tradicionales no es reciente^9^, durante el estallido social fueron duramente criticados por la ciudadanía, al igual que el rol que cumplieron los periodistas en esta crisis^10^. En este contexto, la televisión abierta fue el medio peor evaluado y se puede constatar que en esta etapa los índices de audiencia televisiva cayeron, experimentando un descenso entre octubre y diciembre de ese año^11^. Sin embargo, esta situación se revirtió durante la pandemia, ya que de acuerdo con los datos del Consejo Nacional de Televisión hubo un aumento de consumo de televisión abierta, tanto en número de personas como de cantidad de horas frente a la pantalla, especialmente, durante los períodos de cuarentena^12^. También cabe destacar que la radio fue el medio que contaba con mayor confianza entre la ciudadanía, mientras que las redes sociales (WhatsApp, Facebook, Instagram, Twitter, YouTube e Instagram) si bien fueron diariamente utilizadas, también fueron las peor calificadas^13^.

En cuanto a la comunicación oficial entregada por el Ministerio de Salud, destacan los informes diarios de COVID-19 emitidos por televisión abierta, en los que las autoridades sanitarias desarrollaron una estrategia comunicacional centrada más en la entrega de cifras (contagiados, fallecidos, test, recursos disponibles, etc.) que en explicar las medidas de autocuidado^14^. Además, se desplegaron campañas principalmente por redes sociales y, en menor medida, por otros medios de comunicación. Estos elementos contextuales permiten comprender el escenario en el que se desarrolló la primera etapa de la pandemia en Chile. 

### Sobrecarga informativa

La avalancha informativa que marcó los primeros meses de la pandemia de COVID-19 se caracterizó por la prevalencia de circulación de información técnica de alta complejidad y una gran diversidad en cuanto a la calidad de dicha información, los mensajes y las fuentes informativas. Entre todos los mensajes que circularon en ese periodo coexistieron información científica y rigurosamente generada con mensajes confusos o falsos^4^^,^^15^. Adicionalmente, se debe considerar que la pandemia de COVID-19 fue la primera que se enfrentó en un escenario de alta penetración de medios masivos pero, sobre todo, de medios digitales, a través de los cuales, los caudales de información fluyen con más velocidad y con menos filtros que a través de los medios tradicionales. Ese contexto, de abundancia, complejidad y dudas respecto de la veracidad de los mensajes, pudo ser terreno fértil para que las personas experimentaran sobrecarga informativa.

El concepto de *sobrecarga informativa* parte de la noción de que los individuos tienen una capacidad limitada para percibir, codificar, entender y recordar la información de su entorno^16^. La sobrecarga informativa tiene lugar cuando las demandas del entorno informativo superan las capacidades cognitivas de una persona para procesar la información y se expresa como un estado aversivo en el que las personas se sienten fatigadas y estresadas^17^. Adicionalmente, la sobrecarga se asocia a la aparición de emociones negativas y deseos de descontinuar el uso de fuentes informativas^18^. De hecho, en el contexto de salud, la sobrecarga se ha asociado negativamente con la búsqueda de información a través de Internet^19^ y positivamente con la evitación de contenidos^20^. Recientes indagaciones han mostrado que la sobrecarga informativa, además, influye en la práctica de comportamientos preventivos^21^.

La sobrecarga informativa ha sido estudiada desde diversas disciplinas, sobre todo, desde ciencias de la información, administración, estudios del periodismo y comunicación política, entre muchas otras, y se la asocia con muchos conceptos similares, como sobreabundancia de información, fatiga informativa y sobrecarga cognitiva, entre otros^22^. En comunicación de la salud se ha indagado profusamente acerca de la sobrecarga informativa en el contexto del cáncer; sin embargo, la sobrecarga relativa a otros temas de salud ha sido menos estudiada^23^. Más recientemente, y en el contexto de la pandemia por COVID-19, ha resurgido el interés por el estudio de este concepto en el campo de la comunicación de la salud, mirando más allá de la comunicación del cáncer^24^.

### Sobrecarga y evitación

La evitación corresponde a la realización de esfuerzos activos por ignorar información acerca de un tema que forma parte del entorno informativo de los individuos^25^. Es importante señalar que la evitación va más allá de la ausencia de búsqueda informativa, puesto que también se incluyen en esta conducta esfuerzos para evitar la exposición incidental o escaneo^26^^,^^27^. Estudios previos han mostrado la conexión entre la sobrecarga y la evitación de información^20^^,^^28^, sosteniendo que quienes se sienten sobrecargados tienen mayores probabilidades de evitar contenidos. Desde la perspectiva de la comunicación de la salud resulta central estudiar más en profundidad la evitación informativa y sus causas, debido a que puede tener severas consecuencias para la toma de decisiones en salud de las personas, puesto que, si usan solo parcialmente la información disponible en su entorno, pueden basar sus decisiones en información incompleta. La literatura ha mostrado que la evitación de información puede tomar diversas formas, como la falta de atención, la evitación física o la ausencia de recordación de los contenidos, así como la interpretación sesgada de información^29^. Desde la perspectiva del manejo de la incertidumbre, la evitación de información es un mecanismo para mantener este estado, en contraposición con lo que se ha observado respecto de la búsqueda activa. Mientras esta última es un método para reducir la incertidumbre por el que optan aquellos que se sienten incómodos en dicho estado, la evitación de información es el camino que eligen quienes encuentran en el estado de incertidumbre un ambiente más satisfactorio que el de las certezas^30^^,^^31^.

### Sobrecarga y búsqueda

La búsqueda de información corresponde a esfuerzos activos que hacen los individuos por acceder a información y ha sido un concepto ampliamente estudiado en el campo de la comunicación de la salud, toda vez porque esta conducta informativa se asocia con resultados favorables de salud y con la práctica de comportamientos preventivos^32^^,^^33^^,^^34^. 

En lo que respecta a la relación entre la búsqueda de información y la sobrecarga, la relación se puede establecer en dos niveles. Por un lado, es posible que la búsqueda informativa genere la exposición a un exceso de información contradictoria, confusa o inverosímil y, por ende, dar pie a la sobrecarga informativa. En ese escenario, la búsqueda de información sería un antecedente de la sobrecarga. Evidencia en esa línea fue encontrada por Soroya y colegas^28^, quienes en un estudio entre adultos en Finlandia en el contexto de la pandemia de COVID-19 concluyeron que quienes buscaban información tenían más probabilidades de experimentar sobrecarga. Por otro lado, considerando que la sobrecarga produce una reacción aversiva hacia la obtención de más información, es posible que como consecuencia de la sobrecarga las personas decidan dejar de buscar información o hacerlo en menor medida. Evidencia en esa línea fue encontrada por Jensen *et al*.^35^, quienes muestran que entre las personas con alta sobrecarga informativa la conducta de búsqueda era menor. En la misma línea, Swar *et al*.^19^ encontraron que quienes experimentaban sobrecarga tenían menos intenciones de buscar información de salud.

## METODOLOGÍA

Este estudio utilizó un enfoque cualitativo interpretativo, que permitió abordar las experiencias de los sujetos y las formas en que estos construyen un entorno informativo y conductas a partir de los significados que atribuyen a sus vivencias^36^.

Entre septiembre de 2020 y enero de 2021 se realizaron 90 entrevistas semiestructuradas a personas residentes en las regiones de Valparaíso y Metropolitana, consideradas como las más pobladas y densas del país. Asimismo, estas regiones presentan gran movilidad de población y concentran un alto porcentaje de personas mayores^38^. Se conformó una muestra teórico-intencional^37^, integrada por personas pertenecientes a los siguientes grupos de riesgo por COVID-19: a) personas de 65 años y más; b) personas con diabetes y/o hipertensión mayores de 18 y menores de 65 años (Tabla 1)

**Tabla 1:** Características de la muestra teórico-intencional (n=90). Región Metropolitana y de Valparaíso, Chile. Septiembre 2020 a enero 2021.

Características	n	%
Sexo		
Mujer	46	51,1
Hombre	44	48,9
Grupos de riesgo		
Personas de 65 años y más	45	50,0
Otros grupos de riesgo^1^	45	50,0
Nivel educativo		
Alto	43	47,8
Medio	28	31,1
Bajo	19	21,1
Regiones		
Región Metropolitana	50	55,6
Región de Valparaíso	40	44,4

**Fuente:** Elaboración propia. ^1^Personas con diabetes y/o hipertensión, mayores de 18 y menores de 65 años.

Debido a las restricciones por las condiciones sociosanitarias y con el objetivo de evitar riesgos de contagio por COVID-19, se tomó la decisión de realizar las entrevistas de manera remota, utilizando mayoritariamente videollamadas (a través de plataformas Zoom, WhatsApp y Meet) y también por teléfono, según la voluntad y preferencia de las personas entrevistadas. Si bien este formato de entrevista permitió configurar una muestra equilibrada en cuanto a género, región y grupo de riesgo, quedó sobrerrepresentado el grupo de personas con mayor nivel socioeconómico y educativo, dado su mayor acceso a telefonía y particularmente a Internet, donde además hay una importante brecha según grupos etarios^39^ (Tabla 1). 

La pauta de entrevistas consideró una ficha inicial con datos sociodemográficos de las personas entrevistadas y un cuestionario diseñado a partir de los objetivos específicos de la investigación: 1) conocer cómo las personas se informan sobre la pandemia, 2) examinar la relación entre las variables del entorno informativo y las percepciones de riesgo de los grupos estudiados, 3) establecer relaciones entre las variables anteriores y la adopción y mantención de comportamientos preventivos de la infección por Coronavirus y/o la intención de adoptarlos, y 4) constatar diferencias entre personas de distintos segmentos socioeconómicos en los aspectos abordados. Se utilizaron dos pautas de entrevistas distintas: una para el grupo de personas con enfermedades crónicas mayores de 18 y menores de 65 años; y otra para personas de 65 años y más.

Las entrevistas fueron transcritas íntegramente y todo el material fue codificado utilizando el programa Atlas.ti, a partir de códigos derivados de la misma pauta de entrevista y temas emergentes. Con el objeto de alcanzar acuerdo y coherencia entre codificadores se realizó un proceso de intercodificación^40^, llegando a un total de 137 códigos. El análisis siguió un enfoque inductivo^41^, distinguiendo temas y patrones recurrentes en las citas, que permitieran generar unidades temáticas de significado desde las experiencias de los sujetos^42^^,^^43^, para luego profundizar en aquellas más relevantes según los objetivos de la investigación. A partir del análisis temático se identificaron cuatro temas: 1) la sobrecarga informativa como problemática; 2) la evitación de información como estrategia de autocuidado en mujeres; 3) corroboración de información: búsqueda activa de fuentes confiables; y 4) uso diferenciado de medios: la TV como el medio más usado, pero también más evitado.

### Aspectos éticos

Cabe señalar que todas las personas entrevistadas leyeron y firmaron un consentimiento informado para participar en este estudio y que la investigación EIS-COVID fue aprobada por el Comité de ética de la Universidad Diego Portales, tal como exige la Agencia Nacional de Investigación y Desarrollo de Chile (ANID).

## RESULTADOS

### La sobrecarga informativa como problemática

Durante el primer año de desarrollo de la pandemia, las personas entrevistadas señalaron haber accedido a gran cantidad de información sobre COVID-19, tanto por una mayor exposición a información proveniente de medios de comunicación tradicionales y redes sociales, como por búsquedas activas en internet, principalmente, en sitios como Google.

En este marco, uno de los aspectos más destacados de los resultados del estudio fue la relevancia que adquirió la sobrecarga informativa para gran parte de las personas entrevistadas, quienes expresaron en sus propios términos sentirse “bombardeadas”, “chatas”, “ahogadas” o “cansadas” de “tanta, “excesiva”, “demasiada” información. Esta sobrecarga es relatada como parte de un proceso gradual que va desde una etapa inicial, al comienzo de la pandemia, en la que había mayor incertidumbre pero, a la vez, más predisposición a recibir información, hasta una etapa posterior en la que se indica una saturación informativa.

*…yo antes lo escuchaba cuando recién empezó, los primeros meses, inclusive iba anotando la estadística, si aumentaron los muertos, qué comuna, tenía un cuaderno donde anotaba porque a tal hora daban el informe, pero ahora ya no veo… y cuando me meto y está el informe paso de largo y veo qué se yo, los vikingos, cosas así porque ya empieza como le digo a hastiar un poco.* (Hombre, 78 años, Región de Valparaíso)

*…al principio veía esto de la universidad que iba dando los países, los índices, cuántos contagiados tenía, pero eso me duró unas dos semanas, ya después fue como “noooo” porque ya uno se empieza ah, como dicen ahora, a sicopatear* […] *después uno termina respirando nomás, pero no viviendo poh. Entonces, como que por ese lado, buscábamos como lo justo y necesario, y nada más poh.* (Mujer, 41 años, enfermedad crónica, Región Metropolitana)

*…Yo creo que me saturé, que cuando terminó la cuarentena de fines de julio y principios de agosto dejé de estar pendiente, pero antes, yo escuchaba todos los reportes, pero es que después me saturé,* (Mujer, 68 años, Región Metropolitana)

En este marco, el relato de la sobrecarga informativa iba acompañado de algunas ideas que se repetían de manera transversal respecto al contenido, las fuentes informativas, los canales, los medios, las emociones y sus consecuencias.

Con respecto al contenido, las personas señalaron que los datos oficiales se volvieron repetitivos con el tiempo y, por tanto, dejó de tener sentido acceder a ellos. Adicionalmente, durante el proceso circuló información contradictoria, generando confusión y/o desconfianza y hubo gran cantidad de información que se identificó como posibles *fake news* y/o vinculadas a “teorías conspirativas”.

En lo relativo a las fuentes, en los relatos se destacó la existencia de una gran cantidad de información y su procedencia de múltiples fuentes: oficiales, no oficiales, nacionales, internacionales, expertas, testimoniales, etc. Por una parte, apareció como problemática la posibilidad de verificar estas fuentes y, por otra, se destacó el hecho de que no siempre se confiaba en las fuentes oficiales. En lo que respecta a los canales o medios, se asoció mayor grado de sobrecarga al consumo de televisión y de redes sociales, más que a otros medios como la radio o la prensa.

En referencia a las emociones, las personas relataron que la información de COVID-19, tanto por su contenido como por la cantidad, les generaba emociones negativas como ansiedad, miedo o incertidumbre. En esa misma línea, se identificaron consecuencias atribuidas directamente a la sobrecarga informativa, tales como los problemas de salud mental, el aumento de la incertidumbre debido a que no es posible diferenciar con claridad la información útil de la inútil y la generación de confusión debido a la abundancia de información.

Cabe señalar que la sobrecarga apareció como una problemática transversal, pero las personas desplegaron distintas estrategias para enfrentarla, entre las que destacaron: a) la evitación de información, b) la corroboración de contenidos y búsqueda activa de fuentes confiables, o c) el uso diferenciado de medios.

### La evitación de información como estrategia de autocuidado en mujeres

La sobrecarga informativa fue planteada por personas de ambos géneros, quienes problematizaron no solo la cantidad de información recibida, sino otros dos aspectos: la información repetitiva y aquella considerada como negativa. Sin embargo, en este punto se observa una de las principales diferencias de género ya que, si bien hombres y mujeres mostraron una preocupación por la forma en que la exposición a noticias negativas podía afectar su estado de ánimo y, especialmente, su salud mental, son las mujeres quienes realizaron una marcada asociación entre el tipo de información que recibían y emociones negativas como miedo, ansiedad, angustia e incertidumbre, entre otras.

*Al principio s*í *poh. Por eso me alejé de ver tanta noticia. O sea, estábamos todos los días pendiente: “qué va a decir el ministro hoy día, ahh chuta cuántos se murieron, cuántos se contagiaron”. Entonces al final empiezas a tener miedo, ¿ya? Empiezas a dormir mal. Y eso al final, en vez de ayudar, toda esa información negativa, te afecta poh. Entonces, como yo ya he estado con un cuadro de estrés, un poco de depresión, sobre todo hipertensa… ehh, es una forma de autocuidado… buscar distracciones en vez de enterarme mucho de lo que está pasando.* (Mujer, 62 años, enfermedad crónica, Región de Valparaíso)

Ante esta negatividad, una de las estrategias que más utilizaron las mujeres para enfrentar la sobrecarga fue la *evitación de la información*. Esta estrategia consistía en evitar intencionalmente, ya sea por búsquedas o exposición incidental, acceder a información sobre COVID-19, por ejemplo, dejando de ver noticias relacionadas con la enfermedad, cambiando de canales cuando se hablaba del tema, sintonizando otras emisoras o borrando determinados mensajes de redes sociales.

Las entrevistadas mostraron ser conscientes de utilizar la evitación ante la información negativa, señalando que se trataba de una forma de autocuidado o protección para preservar su salud mental y, a la vez, su salud física, ya que para ellas ambos aspectos estaban estrechamente relacionados. De esta forma, según sus propios términos, al evitar la información negativa también evitaban “*afectar su psiquis*”, “*somatizar*”, “*debilitar su sistema inmune*”, “*bajar sus defensas*”, “*psicosearse*”, entre otros.

*…de todo, la noticia de la noche al principio la veía más para informarme de la gente que estaba contaminada, qué sé yo, en la tele y llegó un momento en que dije “no quiero verlo más” porque hace daño y afecta la psiquis. Así que me alejé, sonará algo egoísta aquí, pero no, decidí que ni la radio ni la televisión era sano para la mente de las personas*. (Mujer, 70 años, Región Metropolitana)

…*tengo que aprender a manejar el tema y que no me afecte, ahí me cuesta un poco, por ejemplo, cuando veo o escucho, cuando empecé a leer esta cuestión de cómo actuaba esta cuestión del COVID,* […] *Entonces nada, olvídate, yo empezaba a somatizar todo y entonces un día en la noche me acuerdo que desperté y que las piernas me tiritaban, me temblaban las manos, me tuve que levantar, me sentía como ahogada, entonces tuve que evitarlo porque me pasa que es inconsciente, no logro solo leer la información y que me sirva o que no me sirva, leerla, informarme, pero es que mi cabeza empieza a sentir toda la tontera que leí. Entonces por eso evité hacerlo*. (Mujer, 48 años, enfermedad crónica, Región Metropolitana)

### Corroboración de información: búsqueda activa de fuentes confiables

Ante la gran cantidad de información circulante, otra de las estrategias utilizadas fue la corroboración de la información que, en palabras de las personas entrevistadas, era considerada “*dudosa*”, “*poco confiable*” o “*posibles fake news*”. Esta corroboración se hacía principalmente a través de búsquedas activas en Internet, seleccionando las fuentes informativas que se percibían como confiables.

Sin embargo, cabe señalar que hubo variaciones en estos aspectos. Por una parte, la búsqueda activa de información sobre salud general era una práctica relevante para quienes tenían enfermedades crónicas y para personas mayores con enfermedades o autopercepción negativa de su salud. Esta práctica se acentuó con la llegada de la pandemia, especialmente, entre quienes tuvieron una alta percepción de riesgo respecto al COVID-19. La mayor diferencia se dio en que el uso de Internet en estas búsquedas activas para corroborar o complementar información fue más relevante para las personas con enfermedades crónicas que para personas de 65 años y más.

*…a través de las redes sociales busco información, no sé, por ejemplo, cuando empiezan las cadenas que enviar no sé una noticia equis y empiezo a buscar sobre ese tema a través de los mismos buscadores, sobre ese tema, a ver qué tan de veras es ¿ya? esa es como la forma que tengo de buscar información, si en las redes llega algo y me parece sospechoso digamos que pudiera no ser verídico empiezo a buscar más detalle.* (Hombre, 54 años, enfermedad crónica, Región de Valparaíso)

Es importante señalar que la noción de *fuentes confiables* presenta variaciones entre las personas entrevistadas. Si bien en su gran mayoría manifiestan tener confianza en la información que entregan profesionales de la salud o expertos, las personas mayores recurren más a la consulta cara a cara o confían en médicos que aparecen en los medios, mientras que para quienes presentan enfermedades crónicas -que en nuestra muestra son menores de 65 años- recurren más a complementar con la práctica de corroboración o búsqueda de información a partir del uso de Internet.

…*bueno, la información principalmente, aunque restringida, se la pido a mi médico. Ahí converso, le hago preguntas y si no quedo satisfecho, le vuelvo a insistir y después verifico todo, busco de nuevo la información*. (Hombre, 48 años, enfermedad crónica, Región de Valparaíso)

*En general busco información desde Google y coloco las cosas que me interesan y ahí voy, voy buscando. En general, lo que busco son informaciones oficiales sí. No así opiniones de las personas, sino de algún tipo de organización que me genere cierto nivel de confianza* […] *opiniones de médicos, por ejemplo, organizaciones como el MINSAL, que también tiene información. Eso, en general, organizaciones internacionales, pero todas relacionadas con salud*. (Hombre, 48 años, enfermedad crónica, Región de Valparaíso)

Para algunas personas, la información confiable es la que proviene de fuentes oficiales, tales como el Ministerio de Salud o sitios vinculados a organismos de salud como la OMS. En cambio, la información que circula por redes sociales sería menos confiable, más asociada a “*fake news*” o “teorías conspirativas”.

*Entonces yo estoy constantemente capacitándome y si bien uno tiene un sinnúmero de bombardeo de información con respecto al COVID, porque tanto en las plataformas sociales, como en la vida misma, el hecho de la pandemia ha hecho que haya mucha información disponible, pero yo me remito a los canales oficiales. O sea, tratar de capacitarme por esos canales y la verdad que leer, por ejemplo, los artículos todas esas cosas que salen en Internet de las redes sociales, la verdad, que pesco poco, porque ya suficiente tengo con la cantidad de información que tengo por los canales oficiales.* (Hombre, 58 años, enfermedad crónica, Región de Valparaíso)

Sin embargo, la idea de las fuentes oficiales como “fuente confiable” varía para aquellas personas que manifiestan visiones generales de desconfianza a la medicina oficial, al gobierno o a los organismos internacionales. Otro aspecto que genera desconfianza es la información que se percibe como confusa o contradictoria de fuentes oficiales o entre fuentes expertas.

…[la información] *Sí, ha sido clara, pero poco creíble poh. Entonces no sacan nada con que sea muy clara pero no es creíble. Uno tiene por un lado lo que dice el ministro de salud, por otro lado, tienes a la presidenta del Colegio Médico que refuta todo. Entonces, bueno, ¿para qué lado estamos remando?* (Hombre, 72 años, Región de Valparaíso)

*…al Ministerio de Salud no lo sigo porque al principio la información era muy dispersa y poco coherente, o sea como que no me daba confianza.* (Mujer, 66 años, Región Metropolitana)

De esta forma, se puede observar que la sobrecarga no solo se relaciona con la gran cantidad de información circulante, sino con la percepción de información repetitiva, contradictoria o poco confiable.

### Uso diferenciado de medios: la televisión abierta (TV) como el medio más usado, pero también más evitado

Las personas entrevistadas señalaron haber utilizado diversos medios para informarse sobre COVID-19 (TV, radio, prensa y redes sociales), haciendo un uso diferenciado de acuerdo con la confianza en estos medios y a la etapa de la pandemia.

Uno de los aspectos que más destaca es el rol e importancia que adquirió la televisión abierta en el contexto de pandemia, sobre todo en sus inicios, ya que tal como se señaló anteriormente, este medio venía de experimentar una baja constante de audiencia, lo que se había acentuado durante la crisis sociopolítica previa conocida como “estallido social” de octubre de 2019^7^. 

La importancia del rol de la televisión en la primera etapa de la pandemia también se verificó en nuestra investigación. Si bien la televisión cobró especial relevancia para las personas mayores, también se destaca su uso en el grupo con enfermedades crónicas. Ambos grupos mencionan haber recibido información sobre las medidas preventivas para evitar el contagio de COVID-19 a través de la TV, entre las que se identifican: el lavado de manos, el uso de mascarilla, el distanciamiento social y quedarse en casa. En este punto, se habla de la TV en general; por ejemplo, se mencionan diversas fuentes y programas y no se individualizan las campañas oficiales realizadas por el Ministerio de Salud para promover esos comportamientos.

…*bueno, yo creo que la información está en la televisión a cada rato y sobre todo el primer tiempo se hizo un bombardeo muy fuerte de información. Eh, obviamente yo he dejado de tocarme la nariz, los ojos con las manos, que eso es un asunto de costumbre que uno tiene que es difícil, pero que uno tiene que evitarlo, y lo otro, es que me lavo continuo las manos y andar con mascarilla cuando uno sale*. (Hombre, 71 años, Región de Valparaíso)

Sin embargo, el hecho de que las personas utilizaron más la TV como medio informativo, no quiere decir que lo hicieron acríticamente. Los datos de este estudio permitieron profundizar en las percepciones de las personas respecto a los diversos usos y sentidos que le dieron a la TV, mostrando que la audiencia no confía ciegamente en lo que aparece en pantalla y, además, constatando que el uso de la TV varió en las distintas etapas de la pandemia. Así, en las entrevistas se observa que a medida que avanzó la pandemia, las personas pasaron a identificar a la TV como un factor que contribuyó a la sobrecarga informativa, asociando este medio con expresiones como “*bombardeo de información*”, “*estamos ‘ahogados’ con tanta información*”, “*hubo un momento en que nos saturamos*”. Frente a esto, una de las estrategias desplegadas por las personas entrevistadas también fue la evitación de este medio. Aquí la referencia a la televisión aparece de la mano de expresiones como *“apagar la tele”* o “*dejar de ver la tele*”, especialmente, frente a información que se considera negativa y también se plantea como una forma de autocuidado y preservación de la salud mental.

*…al principio, cuando comenzó el COVID, los medios nos disparaban por todos lados acerca del COVID, llegó un momento que estaba paranoica, súper nerviosa de lo que estaba pasando y un día dije no, se acabó, no veo más televisión, no voy a ver más cuántos son, en cada momento cuánto están subiendo los casos*. (Mujer, 66 años, Región Metropolitana)

*…al principio era ver todo el día la tele prendida y ver los matinales, o sea, yo hacía las cosas y estaba la tele prendida en una parte, en otra parte. Entonces, tú te ibai informando y escuchabai todos los días al ministro. Eso fue hasta como un mes y medio atrás más o menos, porque ya después apagué la tele. Uno se sicosea con la cuestión poh.* […] *porque uno empieza a escuchar que la gente se enfermó, que empezó a sentir, que no olía, que el gusto y uno empieza “parece que no siento lo que estoy comiendo”. Entonces, yo creo que es como mucha información y le dan vueltas en lo mismo y en lo mismo y en lo mismo.* (Mujer, 46 años, enfermedad crónica, Región Metropolitana)

Evitar la televisión no significó que las personas dejaran de informarse o de utilizar otros medios. En este sentido, es que se constata el uso diferenciado de medios como estrategia frente a la sobrecarga, tal como se puede observar en la siguiente cita:

Entrevistadora: Y a propósito de la información que usted decía, que hay mucha información circulando, ¿usted también ha dejado de leer o dejado de escuchar información?

*Entrevistado: Más que nada he dejado de informarme de la tele, porque de repente, como le digo, repiten las noticias 35 veces al día y creo que no es sano, yo creo que uno tiene que informarse por los canales, o sea, la radio me permite informarme, los diarios me permiten informarme a través de Internet, y creo, creo que con eso es más o menos suficiente, digamos*. (Hombre, 54 años, enfermedad crónica, Región Metropolitana).

Entre los medios que se mencionaron como alternativa a la TV se encuentran, principalmente, la prensa digital y la radio. Las redes generaban mayor desconfianza y estaban más asociadas a la saturación de información. Además, las personas entrevistadas eran conscientes de que en su uso hay una brecha generacional. 

## DISCUSIÓN

Diversos autores han señalado que durante la pandemia de COVID-19 asistimos a una sobrecarga informativa que fue acompañada de otros fenómenos comunicacionales como la infodemia^3^^,^^5^, en un contexto en el que el uso de Internet y redes sociales propició una diseminación de información sin precedentes^44^. Un escenario que también se pudo constatar en esta investigación centrada en personas pertenecientes a grupos de riesgo de COVID-19 (personas de 65 años y más y personas con enfermedades crónicas) en Chile. 

La sobrecarga informativa se presentó como una problemática que se fue incrementando con el paso del tiempo. El análisis muestra la importancia que tuvo la primera etapa de la crisis, momento en el que se constata que, ante la incertidumbre, las personas utilizaron tanto redes sociales como medios tradicionales en un grado mayor al que lo hacían tradicionalmente, con predisposición a exponerse a los mensajes oficiales, como los reportes diarios de COVID-19 que emitía el Ministerio de Salud. Sin embargo, a medida que avanzó la pandemia se evidenció una saturación informativa en la cual las personas comenzaron a percibir los mensajes como repetitivos y, en ocasiones, contradictorios. 

Los resultados del estudio muestran que las personas desarrollaron diversas estrategias para sobrellevar la sobrecarga informativa, entre las que destacan: la evitación de la información, la búsqueda activa para corroborar información mediante fuentes confiables y un uso diferenciado de medios de comunicación. 

Tal como hemos señalado, la literatura ya mostraba que tanto la evitación de información como la búsqueda activa están relacionadas con la sobrecarga informativa^28^ y el estudio también arroja resultados en esta dirección. Así, por una parte, muestra que la evitación fue una de las principales estrategias que utilizaron las personas entrevistadas para enfrentar la sobrecarga, pero además que hay una diferencia de género en su uso. Para las mujeres, la evitación adquiere más relevancia, especialmente para eludir emociones negativas y como forma de cuidado de su salud mental. 

En cuanto a la búsqueda activa, estudios previos observaron que esta práctica podía ser considerada un antecedente de la sobrecarga^28^ o bien que, como consecuencia, la sobrecarga generaría una reacción adversa y llevaría a dejar de buscar información^19^^,^^35^. Sin embargo, los resultados de este estudio también indican otra dirección, ya que la búsqueda activa es utilizada entre quienes acusan sobrecarga como estrategia para corroborar información, especialmente, ante aquella considerada dudosa o *fake news*. Esta práctica cobró más relevancia entre el grupo de las personas con enfermedades crónicas que entre las personas de 65 años y más. 

Por último, cabe destacar que es necesario tener en cuenta las particularidades del contexto chileno para interpretar algunos resultados en cuanto al uso de fuentes y medios considerados como confiables. Los resultados muestran que la TV abierta es un medio que, a pesar del desprestigio que traía de períodos anteriores (sobre todo en el contexto del estallido social) cobró relevancia en la etapa de la pandemia, especialmente, para los grupos de mayor edad. Sin embargo, se evidencia un uso crítico de este medio y también se lo asocia a la saturación de información y a la evitación. Otro aspecto a tener en cuenta es que, si bien había un cierto acuerdo respecto a la confianza que inspiraban los profesionales de la salud como fuentes de información, había mayor diversidad de percepciones frente a otras fuentes consideradas como oficiales, por ejemplo, ante las autoridades del Ministerio de Salud. En este punto, cabe recordar que las autoridades del gobierno partieron administrando la pandemia en un contexto de baja aprobación y crisis social, lo que podría haber influido en la percepción de la población entrevistada. 

## CONCLUSIONES

Los principales resultados de esta investigación pueden ser un aporte para el manejo comunicacional de futuras crisis sociosanitarias, especialmente, aquellas con características similares a la generada por la pandemia de COVID-19. El estudio muestra la relevancia que tuvo la sobrecarga informativa durante la pandemia y la necesidad de revisar cómo los grupos de riesgo (personas con enfermedades crónicas y personas de 65 años y más) desarrollaron estrategias para enfrentarla.

Comprender que la sobrecarga fue un proceso que se dio a medida que transcurrió el tiempo y que estuvo vinculada no solo a la cantidad de información, sino a la saturación por la repetición de los mensajes, lleva a plantear la necesidad de desarrollar estrategias de comunicación a largo plazo, que varíen los mensajes y se adecúen a las nuevas necesidades que van surgiendo. La repetición sin variaciones de la información puede llevar a la saturación y a la evitación de las fuentes oficiales.

Por otra parte, el estudio muestra la necesidad de tener en cuenta la diversidad existente, incluso, en los grupos de riesgo. Tal como se ha señalado, la evitación de información fue una de las estrategias desarrolladas por las personas entrevistadas, pero mucho más tematizadas por mujeres, quienes buscaban preservar su salud mental ante noticias negativas. Este es un aspecto a tener en cuenta a la hora de diseñar estrategias de comunicación, especialmente, en la generación de mensajes alarmistas que puedan provocar miedo o aumentar la ansiedad de la población, debido a que el efecto puede ser contraproducente. Otro aspecto a tener en cuenta dentro de la diversidad existente entre los grupos de riesgo es cómo se entiende qué es una fuente o un medio confiable. En este punto, vemos que aunque la confianza en los profesionales de la salud fue transversal, las personas con enfermedades crónicas mantuvieron estrategias previas para corroborar y complementar información mediante búsquedas activas en Internet. 

Por último, cabe destacar el rol social que tuvo la televisión abierta, especialmente, para los grupos de mayor edad, quienes recurrieron a ella consistentemente para obtener información sobre el devenir de la pandemia. Sin embargo, el estudio también muestra la necesidad de considerar que este medio puede asociarse a la saturación de información y a la evitación. Estos aspectos cobran relevancia a la hora de pensar estrategias de comunicación efectivas para llegar a grupos de riesgo como las personas de 65 años y más, ya que en este caso no puede confiarse solo en el uso de sitios web o redes sociales para el despliegue de campañas oficiales.

En cuanto al desarrollo de futuras investigaciones, consideramos que es necesario tener en cuenta las limitaciones que implicó el desarrollo de las entrevistas por el contexto de la pandemia, debido a que solo pudieron realizarse de manera telemática. En próximas investigaciones es necesario incorporar entrevistas presenciales para evitar los posibles sesgos asociados al uso de tecnologías, ya sea por acceso, nivel socioeconómico o por grupos etarios.
